# Major Histocompatibility Complex Genes as Therapeutic Opportunity for Immune Cold Molecular Cancer Subtypes

**DOI:** 10.1155/2020/8758090

**Published:** 2020-11-17

**Authors:** Paweł Karpiński, Łukasz Łaczmański, Maria M. Sąsiadek

**Affiliations:** ^1^Department of Genetics, Wroclaw Medical University, Wroclaw, Poland; ^2^Laboratory of Genomics & Bioinformatics, Institute of Immunology and Experimental Therapy, Polish Academy of Sciences, Wroclaw, Poland

## Abstract

Current immunotherapies are effective only in a subset of patients, likely due to several factors including defects in tumor cell antigen presentation, decreased response to immune effectors, and molecular heterogeneity of cancers. Recent molecular classifications enable the categorization of many tumor types. However, deregulation of major histocompatibility complex (MHC) gene expression is poorly characterized in the context of molecular cancer subtypes. To suppress the confounding effect of immune infiltrates on expression patterns of immunoregulators, we identified and removed genes with strong correlation to estimated immune compartment levels in each tumor type. Next, we reanalyzed a total of 13 TCGA cancer types encompassing 5651 tumors and 485 normal adjacent tissues by performing unsupervised clustering of 14 MHC genes. Subsequently, resultant clusters were statistically compared in terms of expression of other immune-related genes. Three MHC expression clusters were discovered by unsupervised clustering. We identified concordantly decreased expression of MHC genes (MHC-low) in 26 out of 55 molecular subtypes. Consequently, our study underlines the urgent need for designing strategies to enhance tumor MHC expression that could improve immune cold tumor rejection by cytotoxic T lymphocytes.

## 1. Introduction

Immune cells present in the tumor microenvironment (TME) can either inhibit or enhance tumor growth. Heterogeneity of the TME is determined by the composition of different cell types in the tumor and their activation state, which is regulated by molecular signals to which these cells are exposed [1]. A further layer of complexity is introduced by the remarkable molecular diversity of tumors even within a single cancer type [2]. Tumor cells are able to exploit various mechanisms of immune regulation to suppress activity of immune cells within the TME, thus avoiding antitumor immunity [3]. There is a growing interest in identifying these mechanisms, which may be targeted using immunotherapies (ITs) to enhance tumor rejection by cytotoxic T lymphocytes [4]. ITs which have sparked the most interest involve antibodies to inhibitory immune checkpoint molecules. Among the immune checkpoint inhibitors, PD-1/PD-L1 and CTLA-4 inhibitors showed promising therapeutic outcomes, and some have been approved for certain cancer treatments (e.g., melanoma), while others are under phase III and IV clinical trials [5]. Despite the success of anti-CTLA-4 and anti-PD-1/PD-L1 therapies, current ITs are only effective in a subset of patients, likely due to the molecular heterogeneity of cancer types.

Recent evidence suggests that major immune antitumor responses are driven by T CD4 and T CD8 cell reactivity against two classes of tumor-derived antigens: neoantigens and cancer germline antigen genes (CAGs) [6]. Neoantigens are tumor-specific mutated peptides arising from nonsynonymous mutations. CAGs are genes that are normally expressed in germ cells and aberrantly expressed in a variety of human cancers [7]. Both classes encode highly immunogenic and selective tumor antigens that are now undergoing clinical evaluation for the treatment of a number of solid tumor malignancies by antigen-directed ITs [7].

In general, TMEs can be divided into 3 major phenotypes: immune cold, immune altered, and immune hot [5]. These phenotypes have their own specific mechanisms for preventing immune responses from eradicating tumor cells. Immune cold tumors are characterized by a shortage of T cells in the microenvironment and a lack of suitable T cell activation [8]. Immune altered phenotype exhibits poor T cell infiltration (albeit not absent) due to the presence of multiple chemokines, vascular factors, and a stromal-based barrier. In contrast, immune hot TMEs demonstrate enhanced infiltration of multiple immune cell subtypes and high neoantigen levels, which are counterbalanced by the expression of various immunoinhibitors by tumor cells [8].

With the completion of The Cancer Genome Atlas (TCGA) and subsequent consensus molecular classifications, there is an opportunity for systematic analyses of the various cancer cohorts, including comparisons and contrasts between different disease subtypes [9]. Tumor molecular subtypes represent a heterogeneous set of diseases with diverse pathological and immune features [10]. For example, much research has been conducted recently by a number of groups in order to establish the molecular classification of colorectal cancer [11–13]. This led to identification of four consensus molecular subtypes (CMSs) including highly immunogenic (CMS1), inflamed (CMS4), and two immune cold CMSs (CMS2 and CMS3). In addition to focusing on a single cancer type, a number of research groups have aimed to provide pan-cancer characteristics of the tumor immune microenvironment [14–17]. While these studies profoundly improved the understanding of the molecular and immunological profile of a variety of cancers, much less is known about the immunomodulators' expression with respect to various tumor molecular subtypes. In this study, using 13 TCGA cancer datasets, we aimed to portray major molecular cancer subtypes by overlaying MHC expression clusters together with expression of immunoinhibitors and CAGs, frequencies of nonsynonymous mutations, and levels of T CD4 and T CD8. In this study, we specifically focused on cancer subtypes with a concordant decrease of MHC expression when compared to corresponding normal adjacent tissue.

## 2. Materials and Methods

### 2.1. Data Acquisition

RNA-Seq-based gene transcription profiles (raw counts, Illumina HiSeq) were downloaded from NCI Genomic Data Commons (GDC) using the TCGABiolinks package [18]. Level 1 Illumina 450k data were downloaded from the NCI Genomic Data Commons (GDC) using GDC Data Transfer Tool except for healthy tumor-adjacent stomach tissues which profiles were obtained from Gene Expression Omnibus (accession number: GSE85464). Only primary tumor samples and normal tumor-adjacent tissues were included in subsequent analysis. We included datasets consist of a relatively large number of tumor and normal samples. Consequently, the 13 selected tumor types included bladder urothelial carcinoma (BLCA), breast invasive carcinoma (BRCA), colon adenocarcinoma (COAD), esophageal carcinoma (ESCA), head and neck squamous cell carcinoma (HNSC), liver hepatocellular carcinoma (LIHC), lung adenocarcinoma (LUAD), lung squamous cell carcinoma (LUSC), pancreatic adenocarcinoma (PAAD), prostate adenocarcinoma (PRAD), stomach adenocarcinoma (STAD), thyroid carcinoma (THCA), and uterine corpus endometrial carcinoma (UCEC).

Data on the number of somatic nonsilent mutations (gene level) per sample were calculated based on mutect calls downloaded by TCGABiolinks [18]. Data on the number of somatic nonsilent mutations in 31 MHC genes in MHC-low subtypes were analyzed in the maftools package [19]. CD4 and CD8 T cell relative levels were adopted from Li et al., with the exception of PAAD and ESCA, for which T CD4 and CD8 levels were inferred by the EpiDISH package using a robust partial correlations approach [20, 21]. If possible, the molecular subtype status for samples was adopted from post-TCGA large-scale analyses (BLCA, HNSC, LUAD, LUSC, PAAD, and PRAD); otherwise, samples were annotated by molecular subtype data obtained from corresponding TCGA publications (see Table 1). In order to assess consensus molecular subtypes (CMS) for COAD samples, we used the nearest template prediction (NTP) algorithm implemented in the CMScaller package with default settings using normalized data [22]. Samples with false discovery rate adjusted *p* values > 0.05 were designated “not assigned” and removed from subsequent analysis.

### 2.2. RNA-Seq Data Preprocessing

After filtering of lowly expressed genes, raw counts were normalized using GC-content effect adjustment and quantile normalization [23]. Next, data were normalized using log_2_ (countspermillion + 0.25) transformation [24]. Subsequently, we detected and removed outlier samples by principal component analysis [25]. Prior to supervised batch correction using the ComBat algorithm, we removed duplicated samples or samples representing small batches (≤4 samples) by incorporating plate numbers or tissue source sites as a batch variable [26]. Cancer subtypes represented by less than 10 samples were excluded from downstream analyses.

### 2.3. Illumina 450k Data Preprocessing

Illumina 450k raw methylation underwent quality filtering and was subsequently preprocessed as described previously [2]. Prior to supervised batch correction using the ComBat algorithm, we removed duplicated samples or samples representing small batches (≤4 samples) by incorporating plate numbers as a batch variable.

### 2.4. Deconvolution of Bulk Tumor Methylation Profiles

We used the EpiDISH package to infer fibroblasts and epithelial and immune compartments fractions from corresponding DNA methylation profiles by using a robust partial correlations approach [27].

### 2.5. Selection of Immunomodulators and CAGs

We adopted the list of 162 immunomodulators including 105 cancer germline antigens (CAGs), 25 immunoinhibitors, and 32 MHC genes from Charoentong et al. and Wang et al. [17, 28]. Subsequently, in each cancer type, we identified and removed lowly expressed immunomodulators. Next, we removed immunomodulatory genes with strong (*r* ≥ 0.4) and significant (FDR-adjusted *p* value ≤ 0.05) Spearman correlation with levels of immune compartment (Supplementary Table 1).

### 2.6. Calculation of Relative Effects (Probabilities)

All continuous variables were assessed for distribution (normal, nonnormal) using the Anderson-Darling test. Due to nonnormal distribution of the majority of data, differences between integrative subtypes were calculated by means of permutation-based, nonparametric ANOVA-type statistics implemented in the npmv package [29]. Resultant nonparametric relative effects quantify the tendencies (probabilities) observed in the data in terms of probabilities (0-1 scale). Consequently, relative effects for each variable in each cancer subtype can be described as lower (0-0.4), not changed (0.5), and higher (0.6-1).

### 2.7. Correlation between Selected Immunomodulators

Spearman correlation together with false discovery rate- (FDR-) adjusted *p* values was used to assess mutual correlation of selected immunomodulators in each of the 13 cancer types separately. We defined absolute correlation *r* ≥ 0.4 with FDR ≤ 0.05 as strong. Subsequently, all strong negative and positive correlations detected for each cancer type were converted to -1 and 1 values and summarized to one matrix. All calculations were performed in psych R package.

### 2.8. Unsupervised Clustering

To estimate the number of MHC clusters in our data, we used as input expression probabilities of MHC genes obtained for each cancer subtype by implementing ANOVA-type statistic (see above). Expression probabilities of 14 MHC genes in each cancer subtype were then clustered by the use of the COMMUNAL package using integrative analysis of three clustering algorithms (hierarchical clustering, *k*-means, and pam) and 14 cluster validation measures [30]. We tested a range of clusters (*K*) from 2 to 5. Optimal *K* was defined based on rank aggregation of multiple validation scores. Results of unsupervised clustering were visualized by a heat map [31].

## 3. Results

### 3.1. Selection of Immunomodulators and CAGs

In the present study, we intended to portray expression profiles of selected genes in tumor cells in order to better characterize mechanisms of tumor escape from immune surveillance. Therefore, we aimed to control the confounding effect of levels of immune infiltrates on the expression of immunoregulators and CAGs. Consequently, we used methylation profiles of each tumor and deconvolution algorithm to calculate relative proportion estimates of epithelial, fibroblast, and immune compartment in each tumor. Finally, we decided to discard genes with strong correlation to immune compartment estimates in each tumor type. In brief, out of 162 selected genes, approximately 50% were removed from downstream analyses due to low expression (in at least one cancer subtype) or significant and strong positive correlation with estimated levels of immune compartment of tumors. Therefore, 76 immunomodulatory genes were retained for subsequent analyses including 50 CAGs, 12 immunoinhibitors, and 14 MHC genes. Immunomodulators with strong or weak correlation with estimated levels of immune compartment are listed in Supplementary Table 1. In particular, selected MHC genes included genes involved in antigen processing (*CALR*, *CANX*, *ERAP1*, *ERAP2*, *PDIA3*, *PSMB5*, *PSMB6*, *PSMB7*, *PSMB8*, and *PSMB10*), antigen transport (*TAPPB*), and antigen presentation (*HLA-C*, *HLA-G*, and *HLA-DQA2*).

### 3.2. Correlation between Immunomodulators

**Figure 1:**
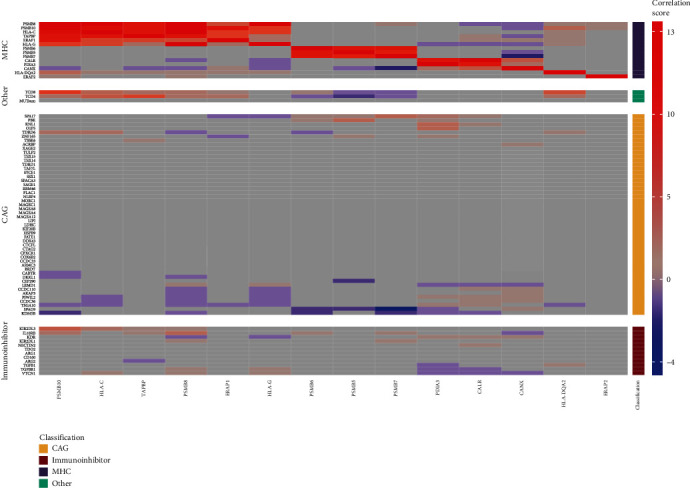
Heat map depicting frequency of correlation of immunomodulators summarized over 13 cancer types. Immunomodulators are split into 4 classes (CAGs, immunoinhibitors, MHC, and other). Correlation of selected immunomodulators in each of the 13 cancer types was calculated separately and summarized (see Materials and Methods). Correlation score reports sum of strong and significant correlations for each variable observed in 13 cancer types. Red denotes positive correlation, gray denotes no significant correlation, and blue denotes negative correlation.

A heat map summarizing the number of strong correlations between immunomodulators in 13 cancer subtypes is provided in Figure 1. We observed mutual, strong, and positive correlations observed in majority of cancer types between *HLA-C*, *HLA-G*, *PSMB8*, *PSMB10*, *TAPBP*, and *ERAP1*. We also observed strong, positive, and mutual correlations between *PSMB5*, *PSMB6*, and *PSMB7*. In addition, *PSMB10* expression was positively correlated with T CD8 levels (in 7 out of 13 datasets) and *TAPPB* was correlated with TCD4 levels in 6 cancer datasets. Other positive correlations were much more variable and were noted in less than 4 cancer datasets. Negative correlations were much less repeatable than negative correlations. The most frequent one was observed between *PSMB7* and *SPAG9* in 4 cancer datasets.

### 3.3. Unsupervised Clustering Uncovers Cancer Subtypes with Low MHC Expression

**Figure 2:**
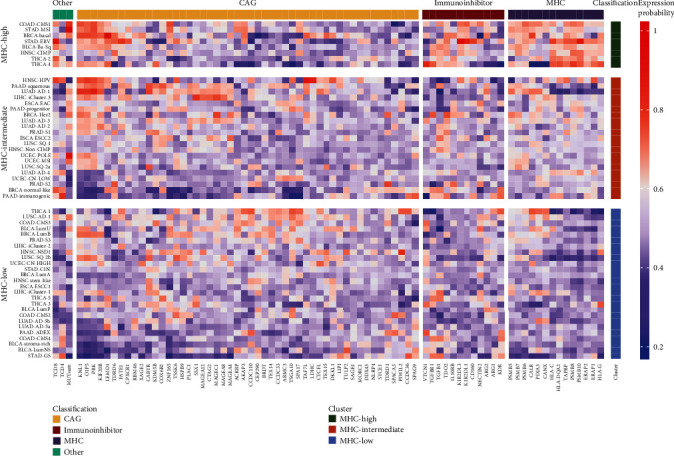
Results of unsupervised clustering of expression probabilities of MHC genes. Major molecular cancer subtypes (*y*-axis) are portrayed by overlaying MHC expression clusters (MHC-low, MHC-intermediate, and MHC-high) together with expression probabilities of immunoinhibitors and CAGs, frequencies of nonsynonymous mutations (other/MUTsum), and levels of T CD4 and T CD8 (other/TCD4 and TCD8). Expression probability for each variable in each cancer subtype can be described as lower (0-0.4), not changed (0.5), and higher (0.6-1).

In the present study, the 5651 tumor samples representing 13 cancer types were assigned to 55 molecular cancer subtypes and corresponding normal adjacent tissues (Table 1). Subsequently, in each cancer type, the gene expression of selected immunomodulatory genes, frequencies of nonsynonymous mutations, and levels of T CD4 and T CD8 were compared between subtypes and normal adjacent tissues by means of multivariate, nonparametric ANOVA-type statistics [29]. Resultant probabilities of each selected variable in each subtype being highly or lowly expressed were collected. Subsequently, expression probabilities of MHC genes in each cancer subtype were subjected to unsupervised clustering using aggregation of 3 major clustering algorithms and 14 validity measures (see Materials and Methods) [30]. Three MHC clusters were proposed as an optimal clustering solution. Consequently, tumor subtypes were classified into three MHC subgroups (Figure 2, Table 1, Supplementary Figure 1): (*i*) with clearly elevated expression of MHC (MHC-high) in 8 cancer subtypes, (*ii*) with intermediate elevation of MHC expression (MHC-intermediate) in 21 cancer subtypes, and (*iii*) with clearly decreased expression of MHC (MHC-low) in 26 cancer subtypes.

### 3.4. Immunoregulatory Correlates of Cancer Subtypes with respect to MHC Expression

We investigated whether MHC subtypes display dependencies that may help to guide personalized immunotherapies. We focused on expression of CAGs, immunoinhibitors, CD4 and CD8 T cell levels, and the number of somatic nonsilent mutations (Figure 1). Supplementary Figures 2–4 depict expression probabilities of each immunomodulator averaged over the three MHC clusters. In general, MHC-high was clearly outstanding in terms of high expression of immunoinhibitors and a higher number of somatic nonsilent mutations and enrichment with CD4 or CD8 T cells (Supplementary Figure 4). Differences between MHC-intermediate and MHC-low were much less pronounced except of significantly lower expression of the majority of MHC genes (Supplementary Figure 1). Expression levels of the majority of immunoinhibitors in MHC-intermediate and MHC-low subgroups were at probability equal to 0.5 indicating lack of change when compared to normal tissue (Supplementary Figure 2). In general, we observed consistent expression of CAGs across 3 MHC subgroups. With few exceptions, most of CAGs were at a similar or lower level of expression when compared to normal tissue. *LEMD1* displayed high expression probabilities in MHC-high. In addition, expression probabilities of *KIF20B*, *PBK*, *OIP5*, and *KNL1* were clearly above neutral threshold (0.5) in MHC-high and MHC-intermediate. *TSGA10* was predominantly elevated in some MHC-low tumors (THCA-1, LUSC-AD1, COAD-CMS3, BLCA-LumU, BLCA-LumP, and COAD-CMS2) (Figure 1, Supplementary Figure 3). MHC-intermediate displayed higher levels of CD8 T cell levels and the number of somatic nonsilent mutations when compared to MHC-low (Figure 1, Supplementary Figure 4). However, there were a few exceptions in the MHC-low subgroup from this trend. For example, BLCA-LumU, LUAD AD-1, and HNSC NSD1 subtypes displayed a high number of nonsilent mutations (Figure 1).

### 3.5. Somatic Mutations of MHC Genes in MHC-Low Subtypes

We investigated the possibility that MHC deficiency in MHC-low subtypes is due to the accumulation of nonsynonymous somatic mutations in MHC genes. We found that the mutation frequency in 31 MHC genes ranges from 0% to 18% depending on cancer subtype (Table 1).

## 4. Discussion

Recently, rapid growth of knowledge has occurred regarding genetic, epigenetic, and proteomic alterations associated with various cancers. This has led to the conclusion that cancer is a heterogeneous disease with molecular alterations often dictating tumor evolution, response to treatment, and outcome [41]. In addition, complex interplay of tumor cells with components of the TME has emerged as a critical aspect of tumor biology and was strongly associated with the host ability to control growth and respond to ITs [42]. Consequently, a number of studies focused on immune subtyping of tumors. For example, Rooney et al. based on pan-cancer analysis of 18 cancer types proposed an RNA-based metric of immune “*cytolytic activity*” and defined several factors that enable tumors to resist immune attack including mutations in antigen presentation machinery [16]. Recently, pan-cancer analysis by Charoentong et al. characterizing immune infiltrates across 20 cancer types provided a multigene predictor (“*immunophenoscore*”) of patient response to checkpoint blockade (CTLA-4 and PD-1 blockers) [17]. Finally, Thorsson et al. in 2018 identified six immune pan-cancer subtypes that are hypothesized to define immune response patterns impacting patient prognosis. This study suggested that certain therapeutic approaches may be considered regardless of tumor location or histology [43]. However, individual tumor types varied substantially in their proportion of immune subtypes and in their prognostic impact [44]. Thorsson et al. also emphasized the importance of CAGs in stimulating T cell responses directed against this antigen class [43].

In parallel to immune subtyping, promising strategies rely on molecular subtyping of tumors, which extends the portfolio of possible effective treatments by characterizing specific biological pathways altered in tumor subtypes. Colorectal cancer with the four subtypes identified (CMS1-CMS4) exemplifies the most robustly characterized cancer type in terms of TME composition and intrinsic pathway alterations [41]. While our analysis is unique in terms of depicting immune landscape of tumor cells in the multiple cancer molecular subtypes, there are several reports characterizing expression of immune-related genes in molecular subtypes of selected tumors [45]. For example, Becht et al. and Karpinski et al. provided independently immune characteristics of consensus molecular subtypes in CRC. In agreement with current analysis, CMS2 and CMS3 were defined as immune cold subtypes with low expression of MHC genes [46, 47]. It has to be noted, however, that in contrast to previous analyses we removed genes strongly correlated with immune compartment of tumor. This was done to depict expression of immune regulators in tumor cell fraction rather than in lymphatic infiltrate. Consequently, some tumor subtypes known for their relatively high levels of immune infiltration were classified as MHC-low (CRC CMS4) or MHC-intermediate (HNSC HPV or PAAD immunogenic).

Due to improved survival and an increased response rate to checkpoint inhibitors, much attention has been paid to cancer subtypes with the immune hot phenotype [8]. However, tumor subtypes representing immune cold phenotypes are much less characterized in terms of possible immunotherapeutic strategies [8]. In this context, the purpose of our study was to specifically depict cancer molecular subtypes using MHC-derived expression clusters and the expression profile of immunomodulators, to indicate possible therapeutic strategies in subtypes displaying the immune cold (MHC-low) phenotype. As mentioned before, we focused on genes which expression was independent from immune infiltration. More than half of selected MHC genes displayed concordant, positive correlation across majority of cancer types. These genes are involved in all critical steps from antigen processing and transport to antigen presentation, thus suggesting existence of biologically important relationships independent of cancer type. Our approach has allowed us to identify a group of 26 tumor subtypes with a concordant decrease of MHC expression when compared to other subtypes and corresponding normal adjacent tissues, which suggests neoantigen processing and presentation dysfunction as a route to escape from T cell-mediated immunosurveillance in these subtypes [48]. Furthermore, all MHC-low subtypes displayed comparable levels of most of immunoinhibitors to normal tissue. This suggests that MHC-low subtypes are unlikely to respond to the majority of current anticancer immunotherapies and trials, including immune checkpoint inhibitors, CAG-based vaccines, and immune-cell engineering [49, 50]. However, low levels of immunoinhibitors also suggest that MHC-low subtypes are unlikely to be infiltrated by anergic and hyperexhausted cytotoxic T cells [51]. Furthermore, elevated expression of some CAGs observed in MHC-low (e.g., *TSGA10*) suggests a weak spot that could be utilized to reactivate T cell-mediated cytotoxicity on condition that the antigen processing and presentation machinery is restored. If we consider the altered pattern of neoantigen (CAGs) expression and infiltration by nondysfunctional cytotoxic T cells observed in most MHC-low subtypes, we find that reconstitution of MHC expression in MHC-low tumors could make them more susceptible to immune elimination. Moreover, the elevated load of somatic mutations observed in BLCA-LumU, LUAD AD-1, and HNSC NSD1 subtypes will make them even more vulnerable to unleashing the preexisting immunity when compared to other MHC-low subtypes [52]. In summary, restoration of MHC gene expression might be very effective in the induction of antitumor immune response in MHC-low tumor subtypes.

It did not escape our attention that MHC-intermediate subtypes defined in this study were relatively similar (in terms of analyzed variables) to MHC-low, except for more elevated expression of MHC genes, expression of four CAGs, and increased incidence of high mutational burden and T CD8 levels. At this stage, we are not able to provide definite interpretation, whether MHC-intermediate tumors will require different therapeutic approach than MHC-low. Further studies including much more variables (for example, miRNA and/or lncRNA expression) are necessary to examine differences between MHC-low and MHC-intermediate subtypes.

Currently, therapeutic approaches against immune cold tumors are very limited. Consequently, immune cold tumors are most challenging to treat and are associated with poor prognosis [8]. In principle, effective treatment of immune cold subtypes will require combinatorial therapies including intratumoral gene therapy (for instance, transfection of the missing MHC using viral vectors) and vaccination to enhance T cell responses [49]. Other promising strategies include bypassing the limitation of HLA-restricted antigen recognition [53]. For example, T cells recruiting bispecific antibodies enable simultaneous binding of a tumor cell surface antigen and the CD3 domain of the TCR complex. Consequently, this recruits the T cells to targeted tumor cells [53]. In numerous preclinical studies, radiotherapy has proven to induce MHC expression at the surface of cancer cells. However, there is still a lack of optimal radiotherapy regimen (in terms of dose, fractionation, sequencing, and timing) to treat immune cold cases [8].

A deeper knowledge on the molecular mechanisms responsible for MHC downregulation in MHC-low subtypes needs to be gained to carefully develop an efficient approach to restore MHC expression [54]. In this study, we did not find evidence that nonsynonymous somatic mutations are responsible for MHC dysfunction in MHC-low subtypes. Therefore, future studies should focus on other potential mechanisms responsible for MHC downregulation.

## 5. Study Limitations

There are potential limitations to this study that should be considered. First, by preforming pan-cancer analysis, we attempted to find general mechanism(s) shared between many cancer types. This leads to the removal of variables that are specific to certain cancer types; therefore, future studies on MHC expression focused on a single cancer type may certainly provide valuable and important insights. Second, our study relies on transcriptomic profiles derived from bulk tumor samples; therefore, intrasample cellular heterogeneity presents important factor that confounds the analysis. For example, we demonstrated that ~50% of selected immunomodulators are likely to be expressed by immune fraction of tumors. Therefore, further studies are necessary to precisely establish MHC gene expression in epithelial fraction of the tumors in each cancer subtype. Consequently, immunohistochemistry measurements to quantify the MHC protein expression are necessary to validate results obtained in this study.

## Figures and Tables

**Table 1:** Characteristics of cancer types included in this study and results of unsupervised clustering of expression probabilities of MHC genes. TCGA cancer type abbreviations are provided in Materials and Methods.

Cancer type	Tumor	Normal adjacent	Molecular subtypes	Molecular subtype source	MHC-low subtypes	% of samples with MHC mutations in molecular subtypes
BLCA	376	17	Ba-Sq; LumNS; LumP; LumU; stroma-rich	[32]	LumNS LumP LumU Stroma-rich	Ba‐Sq = 13; LumNS = 0; LumP = 13; LumU = 12; stroma‐rich = 13
BRCA	1090	99	Basal; Her2; LumA; LumB; normal-like	[33]	LumA LumB	Basal = 7; Her2 = 8; LumA = 4; LumB = 4; normal‐like = 0
COAD	441	33	CMS1; CMS2; CMS3; CMS4	[22]	CMS2 CMS3 CMS4	CMS1 = 63; CMS2 = 4; CMS3 = 18; CMS4 = 14
ESCA	143	5	EAC; ESCC1; ESCC2	[34]	ESCC1	EAC = 18; ESCC1 = 10; ESCC2 = 6
HNSC	462	40	CIMP; HPV; non-CIMP; NSD1; stem-like	[10]	NSD1 Stem-like	CIMP = 22; HPV = 17; non‐CIMP = 12; NSD1 = 7; stem‐like = 9
LIHC	317	46	iCluster-1; iCluster-2; iCluster-3	[35]	iCluster-1 iCluster-2	iCluster‐1 = 12; iCluster‐2 = 11; iCluster‐3 = 22
LUAD	474	57	AD-1; AD-2; AD-3; AD-4; AD-5a; AD-5b	[9]	AD-5a AD-5b	AD‐1 = 8; AD‐2 = 12; AD‐3 = 15; AD‐4 = 7; AD‐5a = 10; AD‐5b = 0
LUSC	447	46	AD-1; SQ-1; SQ-2a; SQ-2b	[9]	AD-1 SQ-2b	AD‐1 = 7; SQ‐1 = 16; SQ‐2a = 13; SQ‐2b = 7
PAAD	146	3	ADEX; immunogenic; progenitor; squamous	[36]	ADEX squamous	ADEX = 0; immunogenic = 0; progenitor = 0; squamous = 0
PRAD	442	41	S1; S2; S3	[37]	S3	S1 = 1; S2 = 0; S3 = 2
STAD	334	24	CIN; EBV; GS; MSI	[38]	CIN GS	CIN = 8; EBV = 9; GS = 0; MSI = 68
THCA	481	49	THCA-1; THCA-2; THCA-3; THCA-4; THCA-5	[39]	THCA-1 THCA-3 THCA-5	THCA‐1 = 0; THCA‐2 = 0; THCA‐3 = 0; THCA‐4 = 2; THCA‐5 = 2
UCEC	498	32	CN-HIGH; CN-LOW; MSI; POLE	[40]	CN_HIGH	CN‐HIGH = 7; CN‐LOW = 4; MSI = 40; POLE = 79

## Data Availability

Our analysis is based on publicly available data.

## Supplementary Materials

Supplementary materials Supplementary figure 1. Box plots depicting expression probabilities of MHC genes averaged across different cancer subtypes that were assigned to 3 MHC clusters (MHC-high, MHC-intermediate and MHC-low). Y-axis defines expression probability for each variable in each cancer subtype can be described as lower (0-0.4), not changed (0.5) and higher (0.6-1). Supplementary figure 2. Box plots depicting expression probabilities of immunoinhibitors averaged across different cancer subtypes that were assigned to 3 MHC clusters (MHC-high, MHC-intermediate and MHC-low). Y-axis defines expression probability for each variable in each cancer subtype can be described as lower (0-0.4), not changed (0.5) and higher (0.6-1). Supplementary figure 3. Box plots depicting expression probabilities of CAGs averaged across different cancer subtypes that were assigned to 3 MHC clusters (MHC-high, MHC-intermediate and MHC-low). Y-axis defines expression probability for each variable in each cancer subtype can be described as lower (0-0.4), not changed (0.5) and higher (0.6-1). Supplementary figure 4. Box plots depicting probabilities of sum of mutations and estimated levels of TCD4 and TCD8 cells averaged across different cancer subtypes that were assigned to 3 MHC clusters (MHC-high, MHC-intermediate and MHC-low). Y-axis defines expression probability for each variable in each cancer subtype can be described as lower (0-0.4), not changed (0.5) and higher (0.6-1). Supplementary table 1. Correlation between expression of immune-related genes and estimated levels of tumor cellular compartments (epithelial, fibroblasts and immune cells). Strong and significant correlation (Yes) was defined as absolute correlation r ≥ 0.4 with FDR ≤ 0.05.
