# Supplementary material for: Major Histocompatibility Complex Genes as Therapeutic Opportunity for Immune Cold Molecular Cancer Subtypes
Source: J Immunol Res. 2020 Nov 17;2020:8758090. doi: 10.1155/2020/8758090 (PMC7685841; doi:10.1155/2020/8758090)
Supplement: Supplementary materials — Supplementary figure 1. Box plots depicting expression probabilities of MHC genes averaged across different cancer subtypes that were assigned to 3 MHC clusters (MHC-high, MHC-intermediate and MHC-low). Y-axis defines expression probability for each variable in each cancer subtype can be described as lower (0-0.4), not changed (0.5) and higher (0.6-1). Supplementary figure 2. Box plots depicting expression probabilities of immunoinhibitors averaged across different cancer subtypes that were assigned to 3 MHC clusters (MHC-high, MHC-intermediate and MHC-low). Y-axis defines expression probability for each variable in each cancer subtype can be described as lower (0-0.4), not changed (0.5) and higher (0.6-1). Supplementary figure 3. Box plots depicting expression probabilities of CAGs averaged across different cancer subtypes that were assigned to 3 MHC clusters (MHC-high, MHC-intermediate and MHC-low). Y-axis defines expression probability for each variable in each cancer subtype can be described as lower (0-0.4), not changed (0.5) and higher (0.6-1). Supplementary figure 4. Box plots depicting probabilities of sum of mutations and estimated levels of TCD4 and TCD8 cells averaged across different cancer subtypes that were assigned to 3 MHC clusters (MHC-high, MHC-intermediate and MHC-low). Y-axis defines expression probability for each variable in each cancer subtype can be described as lower (0-0.4), not changed (0.5) and higher (0.6-1). Supplementary table 1. Correlation between expression of immune-related genes and estimated levels of tumor cellular compartments (epithelial, fibroblasts and immune cells). Strong and significant correlation (Yes) was defined as absolute correlation r ≥ 0.4 with FDR ≤ 0.05. [file 8758090.f1.pdf]

Supplementary figure 1. Box plots depicting expression probabilities of <u>MHC genes</u> averaged across different cancer subtypes that were assigned to 3 MHC clusters (MHC-high, MHC-intermediate and MHC-low). Y-axis defines expression probability for each variable in each cancer subtype can be described as lower (0-0.4), not changed (0.5) and higher (0.6-1).

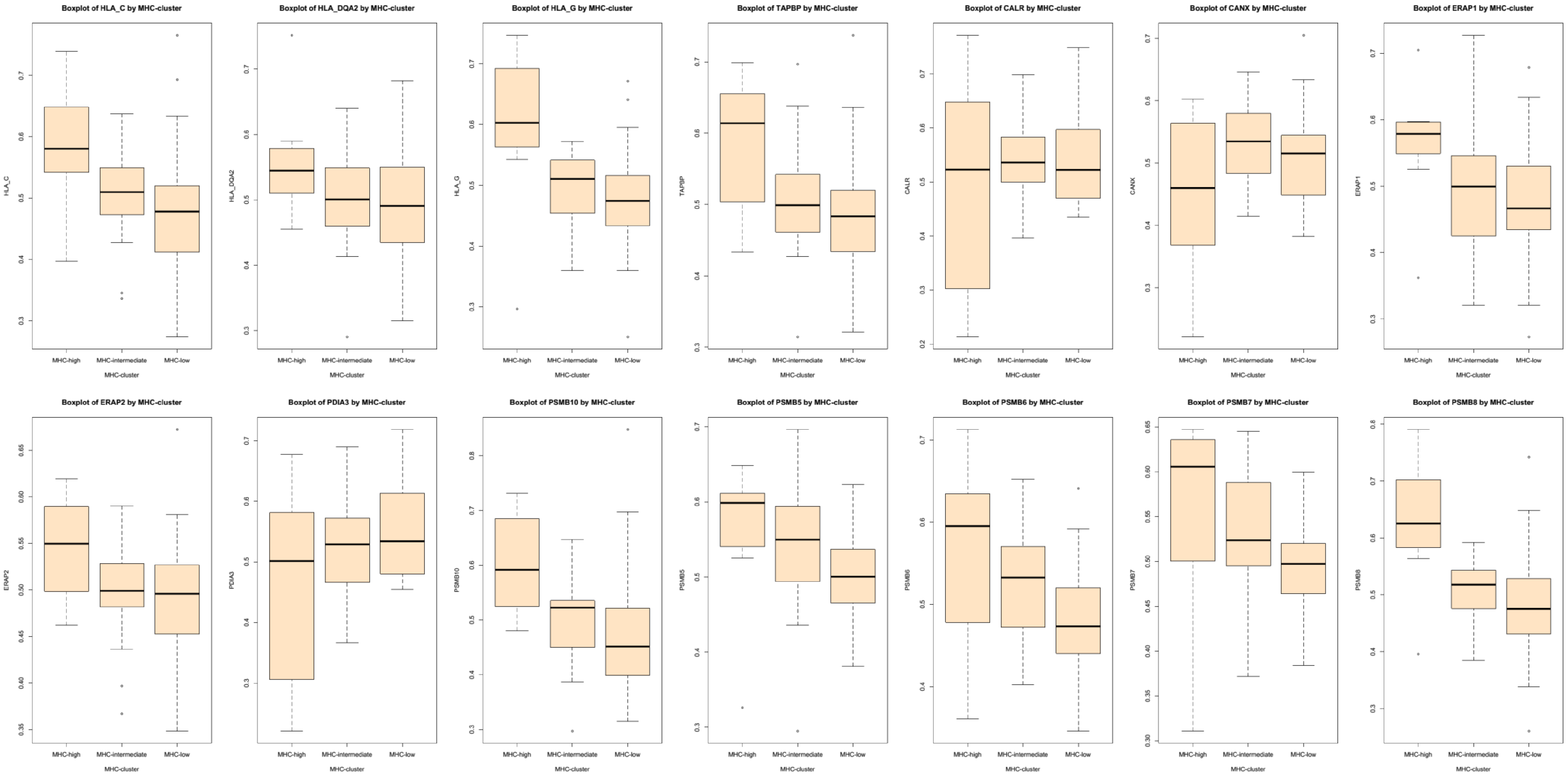

Supplementary figure 2. Box plots depicting expression probabilities of immunoinhibitors averaged across different cancer subtypes that were assigned to 3 MHC clusters (MHC-high, MHC-intermediate and MHC-low). Y-axis defines expression probability for each variable in each cancer subtype can be described as lower (0-0.4), not changed (0.5) and higher (0.6-1).

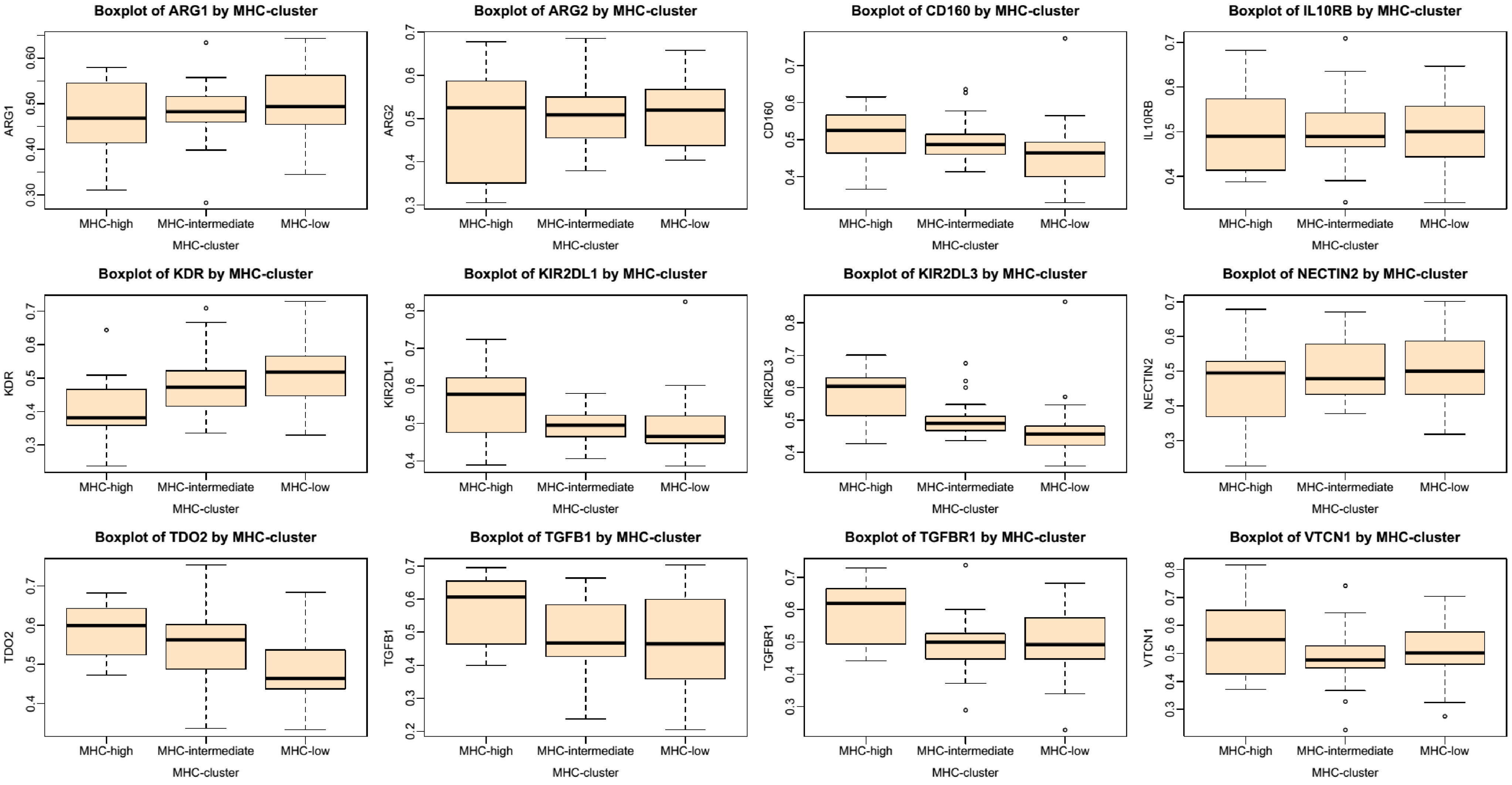

Supplementary figure 3. Box plots depicting expression probabilities of <u>CAGs</u> averaged across different cancer subtypes that were assigned to 3 MHC clusters (MHC-high, MHC-intermediate and MHC-low). Y-axis defines expression probability for each variable in each cancer subtype can be described as lower (0-0.4), not changed (0.5) and higher (0.6-1).

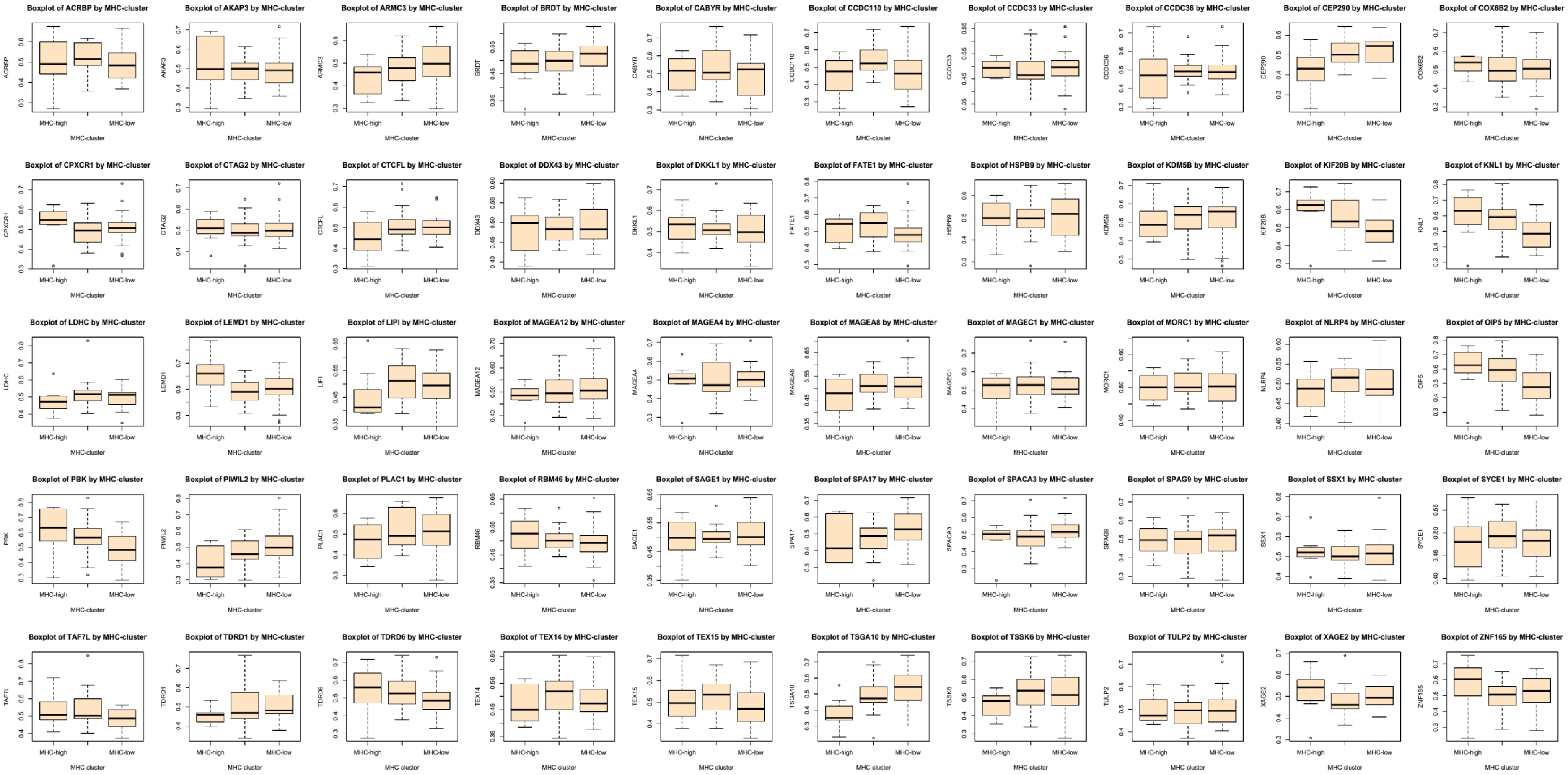

Supplementary figure 4. Box plots depicting probabilities of sum of mutations and estimated levels of TCD4 and TCD8 cells averaged across different cancer subtypes that were assigned to 3 MHC clusters (MHC-high, MHC-intermediate and MHC-low). Y-axis defines expression probability for each variable in each cancer subtype can be described as lower (0-0.4), not changed (0.5) and higher (0.6-1).

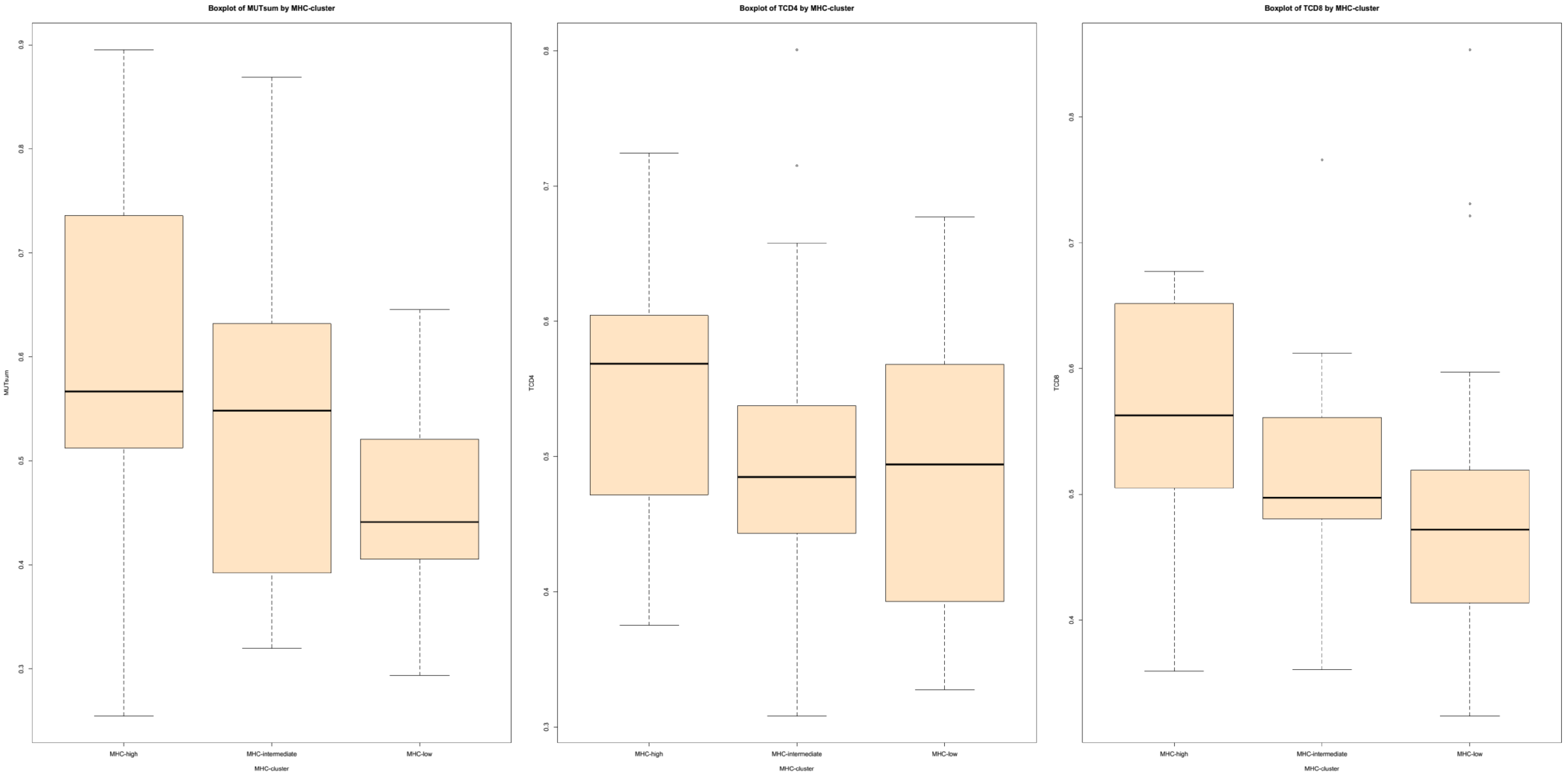

Supplementary table 1. Correlation between expression of immune-related genes and estimated levels of tumor cellular compartments (epithelial, fibroblasts and immune cells) . Strong and significant correlation (Yes) was defined as absolute correlation r ≥ 0.4 with FDR ≤ 0.05.

Gene symbol	Classification	Correlation with immune compartment of tumor
ACRBP	CAG	No
ACTL8	CAG	**Yes**
ADAM2	CAG	**Yes**
ADAM29	CAG	**Yes**
AKAP3	CAG	No
ARMC3	CAG	No
BRDT	CAG	No
CABYR	CAG	No
CCDC110	CAG	No
CCDC33	CAG	No
CCDC36	CAG	No
CEP290	CAG	No
COX6B2	CAG	No
CPXCR1	CAG	No
CRISP2	CAG	**Yes**
CSAG2	CAG	**Yes**
CT55	CAG	**Yes**
CT83	CAG	**Yes**
CTAG1A	CAG	**Yes**
CTAG1B	CAG	**Yes**
CTAG2	CAG	No
CTAGE5	CAG	**Yes**
CTCFL	CAG	No
DDX43	CAG	No
DDX53	CAG	**Yes**
DKKL1	CAG	No
DSCR8	CAG	**Yes**
FATE1	CAG	No
FMR1NB	CAG	**Yes**

FTHL17	CAG	**Yes**
HSPB9	CAG	No
KDM5B	CAG	No
KIF20B	CAG	No
KNL1	CAG	No
LDHC	CAG	No
LEMD1	CAG	No
LIPI	CAG	No
LUZP4	CAG	**Yes**
MAGEA1	CAG	**Yes**
MAGEA10	CAG	**Yes**
MAGEA11	CAG	**Yes**
MAGEA12	CAG	No
MAGEA2	CAG	**Yes**
MAGEA2B	CAG	**Yes**
MAGEA3	CAG	**Yes**
MAGEA4	CAG	No
MAGEA6	CAG	**Yes**
MAGEA8	CAG	No
MAGEB1	CAG	**Yes**
MAGEB2	CAG	**Yes**
MAGEB3	CAG	**Yes**
MAGEB4	CAG	**Yes**
MAGEB5	CAG	**Yes**
MAGEB6	CAG	**Yes**
MAGEC1	CAG	No
MORC1	CAG	No
NLRP4	CAG	No
NXF2	CAG	**Yes**
OIP5	CAG	No
PAGE2	CAG	**Yes**
PAGE2B	CAG	**Yes**
PAGE4	CAG	**Yes**
PAGE5	CAG	**Yes**

PBK	CAG	No
PIWIL2	CAG	No
PLAC1	CAG	No
PRSS54	CAG	**Yes**
RBM46	CAG	No
ROPN1	CAG	**Yes**
SAGE1	CAG	No
SLCO6A1	CAG	**Yes**
SPA17	CAG	No
SPACA3	CAG	No
SPAG9	CAG	No
SPANXA1	CAG	**Yes**
SPANXA2	CAG	**Yes**
SPANXB1	CAG	**Yes**
SPANXC	CAG	**Yes**
SPANXD	CAG	**Yes**
SPANXN5	CAG	**Yes**
SPO11	CAG	**Yes**
SSX1	CAG	No
SSX2	CAG	**Yes**
SSX3	CAG	**Yes**
SSX4	CAG	**Yes**
SYCE1	CAG	No
SYCP1	CAG	**Yes**
TAF7L	CAG	No
TDRD1	CAG	No
TDRD6	CAG	No
TEX14	CAG	No
TEX15	CAG	No
TFDP3	CAG	**Yes**
THEG	CAG	**Yes**
TPTE	CAG	**Yes**
TSGA10	CAG	No
TSPY3	CAG	**Yes**

TSSK6	CAG	No
TULP2	CAG	No
XAGE1A	CAG	**Yes**
XAGE1B	CAG	**Yes**
XAGE2	CAG	No
XAGE3	CAG	**Yes**
XAGE5	CAG	**Yes**
ZNF165	CAG	No
ADORA2A	Immunoinhibitor	**Yes**
ARG1	Immunoinhibitor	No
ARG2	Immunoinhibitor	No
BTLA	Immunoinhibitor	**Yes**
CD160	Immunoinhibitor	No
CD244	Immunoinhibitor	**Yes**
CD96	Immunoinhibitor	**Yes**
CSF1R	Immunoinhibitor	**Yes**
CTLA4	Immunoinhibitor	**Yes**
HAVCR2	Immunoinhibitor	**Yes**
IDO1	Immunoinhibitor	**Yes**
IL10	Immunoinhibitor	**Yes**
IL10RB	Immunoinhibitor	No
KDR	Immunoinhibitor	No
KIR2DL1	Immunoinhibitor	No
KIR2DL3	Immunoinhibitor	No
LAG3	Immunoinhibitor	**Yes**
LGALS9	Immunoinhibitor	**Yes**
NECTIN2	Immunoinhibitor	No
PDCD1	Immunoinhibitor	**Yes**
TDO2	Immunoinhibitor	No
TGFB1	Immunoinhibitor	No
TGFBR1	Immunoinhibitor	No
TIGIT	Immunoinhibitor	**Yes**
VTCN1	Immunoinhibitor	No
B2M	MHC	**Yes**

CALR	MHC	No
CANX	MHC	No
ERAP1	MHC	No
ERAP2	MHC	No
HLA-A	MHC	**Yes**
HLA-B	MHC	**Yes**
HLA-C	MHC	No
HLA-DMA	MHC	**Yes**
HLA-DMB	MHC	**Yes**
HLA-DOA	MHC	**Yes**
HLA-DOB	MHC	**Yes**
HLA-DPA1	MHC	**Yes**
HLA-DPB1	MHC	**Yes**
HLA-DQA1	MHC	**Yes**
HLA-DQA2	MHC	No
HLA-DQB1	MHC	**Yes**
HLA-DRA	MHC	**Yes**
HLA-DRB1	MHC	**Yes**
HLA-E	MHC	**Yes**
HLA-F	MHC	**Yes**
HLA-G	MHC	No
PDIA3	MHC	No
PSMB10	MHC	No
PSMB5	MHC	No
PSMB6	MHC	No
PSMB7	MHC	No
PSMB8	MHC	No
PSMB9	MHC	**Yes**
TAP1	MHC	**Yes**
TAP2	MHC	**Yes**
TAPBP	MHC	No
